# EMERGING STRATEGIES FOR AGING

**DOI:** 10.1093/geroni/igae098.0734

**Published:** 2024-12-31

**Authors:** Fabrisia Ambrosio

**Affiliations:** Chair

## Abstract

This session on Emerging Strategies for Aging focuses on the potential of therapeutically targeting specific pathways/hallmarks of aging for extending healthspan. These targets include increasing mitophagy as a way to improve mitochondrial function, the use of extracellular vesicles from young serum to improve tissue homeostasis, modulating lipid metabolism to reduce the number of senescent cells through induction of ferroptosis and targeting the N6-methyldeoxyadenosine epigenetic mark regulated by DNA damage and stress. The session also will include discussion of approaches to target these different pathways in mice, C. elegans and human cell types derived from iPSCs as well as human brain tissue and provide evidence that genetic variants in lipid metabolism indeed affects the level of senescence in humans. Overall, the goal of the session is to provide examples of the types of novel approaches that could be used to target different pathways important for aging and disease.

